# Advancing Our Understanding of Corneal Herpes Simplex Virus-1 Immune Evasion Mechanisms and Future Therapeutics

**DOI:** 10.3390/v13091856

**Published:** 2021-09-17

**Authors:** Emily Greenan, Sophie Gallagher, Rana Khalil, Conor C. Murphy, Joan Ní Gabhann-Dromgoole

**Affiliations:** 1 Department of Ophthalmology, Royal College of Surgeons in Ireland, D02 XK51 Dublin, Ireland; emilygreenan@rcsi.ie (E.G.); conorcmurphy@rcsi.ie (C.C.M.); 2 School of Pharmacy and Biomolecular Sciences (PBS), RSCI Research Institute, Royal College of Surgeons in Ireland, D02 XK51 Dublin, Ireland; ranakhalil@rcsi.ie; 3 School of Biological and Health Sciences, Technological University (TU) Dublin, Kevin Street, D02 XK51 Dublin, Ireland; c17339893@mytudublin.ie; 4 Department of Ophthalmology, Royal Victoria Eye and Ear Hospital, D02 XK51 Dublin, Ireland

**Keywords:** HSV-1, virus, host-virus interaction, viral replication and pathogenesis, viral immune evasion, antiviral strategies

## Abstract

Herpes stromal keratitis (HSK) is a disease that commonly affects the cornea and external eye and is caused by Herpes Simplex Virus type 1 (HSV-1). This virus infects approximately 66% of people worldwide; however, only a small portion of these people will develop symptoms in their lifetime. There is no cure or vaccine available for HSV-1; however, there are treatments available that aim to control the inflammation caused by the virus and prevent its recurrence. While these treatments are beneficial to those suffering with HSK, there is a need for more effective treatments to minimise the need for topical steroids, which can have harmful effects, and to prevent bouts of disease reactivation, which can lead to progressive corneal scarring and visual impairment. This review details the current understanding of HSV-1 infection and discusses potential novel treatment options including microRNAs, TLRs, mAbs, and aptamers.

## 1. Background to the Disease

Herpes stromal keratitis (HSK) is an ocular disease that is caused by herpes simplex virus type-1 (HSV-1). It is a major cause of infectious corneal blindness in the developed world [1]. HSV-1 is a member of the Herpesviridae family of viruses. This is a family of double-stranded deoxyribonucleic acid (DNA) viruses that is made up of three main subgroups: Alphaherpesvirinae, Betaherpesvirinae, and Gammaherpesvirinae. HSV-1 belongs to the alpha subgroup, which also contains herpes simplex virus type-2 (HSV-2) and varicella-zoster virus (VZV) [2]. The majority of people in the world are infected with HSV-1 [3]. However, more often, ocular involvement results from a reactivation of the virus from its dormant state in the trigeminal ganglion, having tracked in a retrograde fashion to the ganglion from the lip along the mandibular branch of the trigeminal nerve. The virus can establish latency within the host for extended periods of time [4]. There are a number of factors that can cause the virus to reactivate after a period of latency, including trauma, stress, fever, and sunburn [5]. HSV-1 can cause disease in nearly all major ocular tissues including the cornea, eyelids, conjunctiva, and very rarely, the retina. During active infection, lesions can cause transmission of the virus to those who come into direct contact with them via the tears for HSK and saliva for labial HSV-1 infection. The virus is highly transmissible in this way from both symptomatic or asymptomatic individuals [6,7]. HSK is a predominantly unilateral disease. Bilateral cases make up only 1.3–12% of the total and are often associated with immunocompromise [8]. HSV-1 infection of the eye most commonly affects the eyelids, conjunctiva, or cornea [9].

### 1.1. Epidemiology

Studies have shown that the majority of the world’s population has been infected with HSV-1, with the global incidence of HSK is estimated to be 1.5 million [10,11,12]. Ocular HSV-1 is the primary cause of corneal blindness in the developed world due to its recurrent nature [13]. The disease has a significant impact on quality of life due to its impact on visual acuity and the frequency of disease relapses [14].

### 1.2. Symptoms and Diagnosis

**Figure 1:**
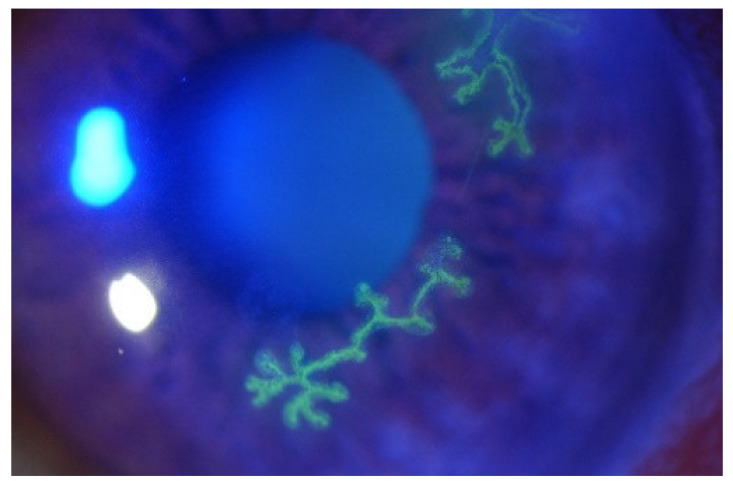
The image shows dendritic lesions caused by ocular HSV infection. The branches of the lesions can be clearly seen, as they have been fluorescently dyed.

Patients with corneal HSV-1 infections typically present with symptoms of discomfort, irritation, redness, watering, and swelling of the eyelid. Patients may also experience photophobia, blurred vison, and pain [15]. Symptoms are usually the most severe in the first 2–3 weeks before improving, although early initiation of antiviral medication can lessen disease severity and course. The diagnosis of ocular HSV-1 is primarily based on the clinical slit lamp examination of the eye [15]. Epithelial disease usually presents as a dendritic lesion located on the corneal epithelium [16]. These lesions contain live virus in the terminal bulbs located at the end of the branched portion, forming the pathognomonic lesion, as shown in Figure 1. When stained with fluorescent dye, the ulcerated lesions can be clearly seen in green. These lesions can enlarge and coalesce to form large, geographic ulcers. Inflammation in the corneal stroma and endothelium may also occur, with subsequent neovascularisation and oedema of the cornea [9,17,18,19]. Recurrences of HSK can lead to progressive corneal scaring and irreversible visual impairment. Damage to the sensory nerves of the cornea can lead to neurotrophic keratopathy, which in severe cases may cause corneal perforation [13].

Polymerase chain reaction (PCR) of a corneal or tear film swab can be used to confirm the diagnosis through the detection of HSV-1 DNA [20]. Another method of diagnosis that may be useful is enzyme-linked immunosorbent assay (ELISA), which can test for the presence of HSV-1 antibodies in serum samples [21]. More recently, the possibility of using microRNAs to aid in viral infection diagnosis has been studied, and evidence suggests certain miRNAs could act as biomarkers for viral diseases, such as Dengue virus [22]. This area is still relatively new, so further research is required to investigate its full potential.

## 2. HSV-1 Replication Strategies and Mechanisms of Pathogenesis

**Figure 2:**
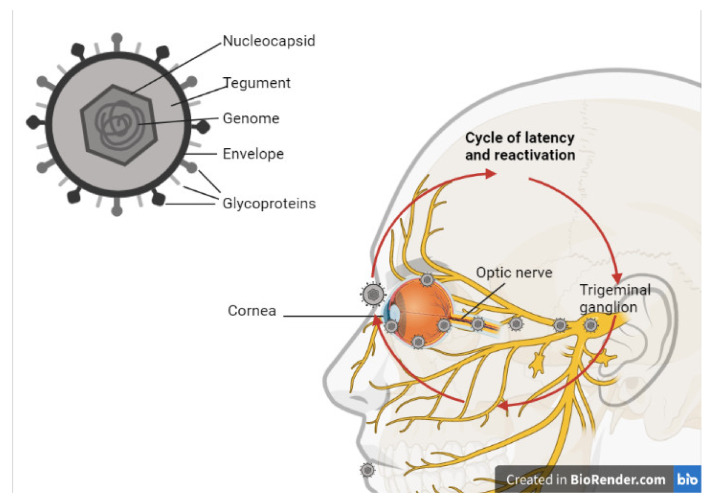
Schematic illustration of HSV-1 virion and the cycle of latency and infection. Created with BioRender.com (https://biorender.com/, accessed on 22 June 2021).

HSV-1 is a DNA virus, its viral particle contains double-stranded linear DNA. It also contains an icosahedral nucleocapsid with a diameter of approximately 100–110 nm where the viral genome is located. This genome is approximately 152 kb in size [23]. Surrounding this nucleocapsid is a tegument, which is made up of proteins and messenger RNAs that allow the virus to evade the immune response of the host and facilitate its replication once it has infiltrated the host cells [24,25]. Then, this is encased within an envelope composed of a lipid bilayer, which contains virally encoded glycoprotein spikes that are important for viral entry (Figure 2). At least 12 different glycoproteins (g) with diverse shapes and sizes have been reported on the surface of HSV-1 to date, including gB, gC, gD, gE, gG, gH, gI, gJ, gK, gL, gM, and gN. These viral glycoproteins facilitate viral entry into the host cells by interacting with receptors on the host cell surface (viral attachment) [13]. Additionally, these glycoproteins interact with other glycoproteins and fuse the viral envelope with the host cell membrane, enabling the delivery of viral content in the cell.The tegument layer consists of approximately 20 viral proteins that are mostly required very early during the infection, including the transcription transactivator VP16 and a protein that induces host mRNA degradation UL41 (known as the virion-induced host shutoff protein, VHS) [25,26]. It has been reported for in vitro studies in cell lines that the presence of four glycoproteins (gB, gD, gH, and gL) is necessary and essential for the delivery of viral content [27].

### 2.1. HSV-1 Host Cell Entry

There is significant interest in understanding the mechanisms and processes involved in HSV-1 entry into cells. The ability to block this step holds the potential to prevent the entry of HSV-1 into host cells and inhibit the virus–host cell interactions that promote viral replication, latency, or cell death.

HSV entry into mammalian cells requires the presence of a specific cell surface receptor able to bind envelope glycoprotein D (gD) and trigger the mechanism leading to membrane fusion [28].

To date, receptors from three structurally unrelated molecular families that facilitate viral entry and the spread of HSV-1 have been identified: Herpesvirus Entry Mediator (HVEM) [29], nectins [30] and heparan sulfate proteoglycans (HSPGs) [31]. HVEM, one of the first identified gD receptors [29], belongs to the TNF receptor superfamily responsible for regulating the host’s immune responses [32]. HVEM is expressed in a wide variety of cell types, including innate immune cell (e.g., NK cell, macrophages), adaptive immune cells (e.g., T cells, B cells, DC), fibroblasts, neurons, and epithelial cells [33]. It has been reported that an induced expression of HVEM in HSV-resistant Chinese hamster ovary (CHO) cells makes them susceptible to HSV-1 infection [29].

In contrast to in vitro studies in clinical isolates conducted by Krummenacher et al. which suggest that HSV-1 can utilise both HVEM and nectin-1 for viral entry [34], more recent studies using HSV-1 gD mutants have suggested that HVEM is not the primary receptor mediating HSV-1 entry into the cornea [33]. Edwards et al. suggested that nectin-1 may instead facilitate the initial entry of HSV-1 on the ocular surface [33].

Nectins are type I transmembrane glycoproteins, belonging to the immunoglobulin superfamily that are expressed by variety of human tissues and cell lines [28]. It has been suggested that HSV-1 binds to both HVEM and nectin-1, with these interactions being vital for HSV-1 corneal infection. However, there is some evidence to suggest that nectin-1 is more efficient at promoting entry compared to HVEM [34].

Using gD with amino acid substitutions, Manoj et al. determined that nectin-1 and HVEM bind different regions of the gD receptor [35]. Furthermore, they determined that nectins are the principal entry receptors for human neuronal and epithelial cell lines, whereas HVEM or nectins could be used to mediate entry into T lymphocyte cell lines [35].

It may be that nectin-1 is the preferred host cell gD recptor for HSV-1, while HVEM has alternate functions. In support of this, studies by Edwards et al. have reported an overexpression of HVEM in corneal tissue only that occurs following HSV-1 infection. This increased expression leads to HVEM-dependent cytokine production from macrophages that contribute to inflammation and loss of corneal sensitivity [33].

In keeping with the suggestion that HVEM may play a more important function during HSV-1 infection, a role for HVEM has been determined during latency and reactivation [36,37]. Allen et al. have shown in Hvem^(−/−)^ mice that the Latency-Associated Transcript (LAT) upregulates HVEM expression, resulting in diminished host immune responses [37].

Furthermore, they found that a lack of HVEM affects latency reactivation but not primary infection in ocularly infected mice [36]. This supports the suggestion that the primary role for HVEM is interaction with the HSV-1 LAT during viral latency and reactivation.

HSV-1 gD has also been shown to bind with 3-O-sulfated heparan sulfate proteoglycan (3-OS-HS) [31]. HSV-1 attaches to 3-OS-HS through viral glycoproteins gB and/or gC [31]. Through a process termed viral surfing, HSV-1 then slides on the cell surface towards the cell body [38]. Then, it binds with cell membrane receptors using gD, gH/gL, and gB glycoproteins, which triggers direct membrane fusion. The absence of HSPGs on the cell surface significantly reduces the HSV-1 infection in murine fibroblast cell lines [39]. Meanwhile, soluble 3-OS-HS or the extrinsic expression of 3-OS-HS enables the infection of HSV-1-resistant CHO cells [40,41].

Supporting the key role of 3-OS-HS in facilitating HSV-1 viral entry, the treatment of cells with heparanase, which cleaves HS chains, has been shown to inhibit HSV-1 entry in mouse explant models [42].

Further research is required to tease out the interplay between 3-OS-HS, HVEM, and nectin-1 and the requirement or preference of HSV-1 for their use for viral entry, latency, and reactivation.

In addition to the pH-independent HSV-1 entry mechanisms described, HSV-1 can also enter the host cell through pH-dependent endocytic pathways. The mild acidic pH in the endosome has been shown to support the activation of viral glycoproteins [43]. While the process that initiates internalisation has yet to be determined, this pH-dependent entry mechanism has been described as an atypical endocytic pathway that has features of non-professional phagocytosis [44].

During pH-dependent viral entry, HSV-1 virions are internalised and transported into the host cell’s early endosomes, where the mild acidic pH of the endosome induces conformational changes in the viral fusion proteins. These conformational changes enable the viral envelope to fuse with the vesicular membrane, which releases the nucleocapsid and tegument protein from the vesicle into the cytosol [45].

### 2.2. HSV-1 Cycle of Latency and Infection

Following infection with HSV-1, the virus establishes latency in the sensory neurons of an infected host [46]. Studies in humans and rabbits have found that the latent virus can spontaneously reactivate and travel back to the original site of infection, resulting in recurrent disease in which free virus can be detected in the tears [47,48]. Interestingly, the spontaneous reactivation of HSV-1 has not been reported in mice, although the finding of lytic transcripts in latently infected ganglia has led to suggestions that subclinical reactivation may be occurring [49].

During a primary ocular infection, the eye is infected at the corneal epithelium, where the virus can then enter the host cells and cause lysis. This primary infection may not cause symptoms, but it can allow the virus to replicate by infecting the adjacent cells [50]. Then, the virus can establish latency by moving into the trigeminal ganglion, where it can remain for the lifetime of the patient. This process is shown in Figure 2. If reactivation occurs, anterograde transport occurs along the ophthalmic nerve, and the infection can reoccur in the eye [51]. HSV-1 differs from retroviruses in that it has the ability to store its genome within the host cell nucleus, allowing episomal latency. While the virus is in its latent state, LATs are produced.

The establishment of HSV-1 latency is associated with the expression of LATs in every neuron, while only 30% of these cells are producing LATs at a given time [52]. Although LAT function is not entirely understood, it has been suggested that they are involved in the regulation of latency via translational or transcriptional repression and play a role in keeping the genome of the virus compact, which allows the possibility of reactivation in the future [51,53,54]. This reactivation leads to recurrent infections that are difficult to treat [55]. A more common route of infection for HSV-1 is via the oral mucosa. From here, the nucleocapsid can translocate to the trigeminal ganglia, where the virus can establish latency. If reactivation of the virus is triggered by any of the common factors responsible for reactivation, an ocular infection can occur, even if the original viral route of entry was not the eyes [56].

**Figure 3:**
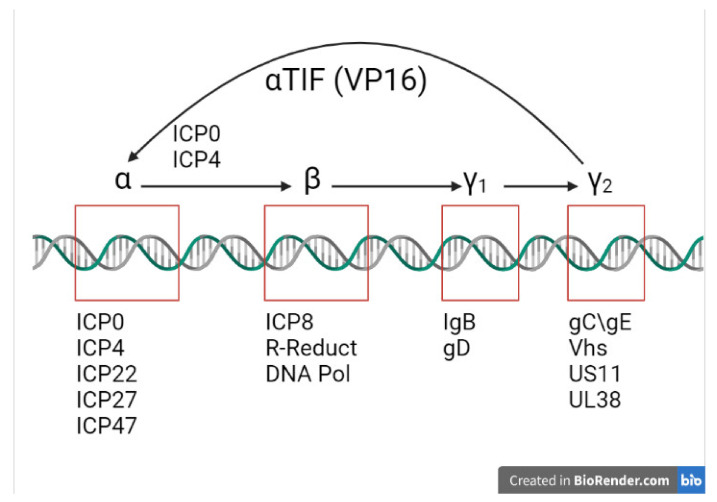
Schematic showing the activation of HSV-1 viral gene expression. VP16 upregulates α genes. ICP0 and ICP4 lead to the expression of α (immediate early) and β (early) genes. γ1 (leaky late) and γ2 (true leaky) gene expression requires viral DNA synthesis and ICP22 and ICP27 (modified from Roizman et al., 2005) [60]. Created with BioRender.com (https://biorender.com/, accessed on 5 July 2021).

HSV-1 attaches to the host cell membrane through a successful interaction between the virion envelope glycoproteins and host cell surface receptors [57]. The fusion of the membranes (also known as adsorption) is aided by the viral glycoproteins gB and gH, while gD is required for entry of the virus [58]. Then, the nucleocapsid is released into the cytoplasm, and the viral DNA is transported into the host cell nucleus via the binding of the tegument proteins to the cytoskeleton microtubules [59]. The entry of viral DNA signals a coordinated and sequential cascade of transcription that leads to the expression of all classes of viral genes required for successful replication, as illustrated in Figure 3.

### 2.3. Immediate Early Proteins

IE (Immediate Early (IE)) or α genes are the first to be expressed by the HSV virus as it enters an infected cell, leading to the production of corresponding proteins. The expression of IE genes is driven by the virion tegument protein, VP 16 [61], which forms a tri-partite complex with two other cellular factors, OCT-1 and HCF, to trigger the expression of IE genes. This marks the IE stage of the lytic phase of the HSV-1 viral life cycle. Five different IE gene products, the infected cell proteins (ICP), have been identified: ICP0, ICP4, ICP22, ICP27, and ICP47 [61,62,63,64]. At least four of these are implicated in the transcription of the remainder of the viral proteins in the following Early (E) and Late (L) phases. The expression peak of IE genes is at 2 to 4 h post infection, although the production of most IE proteins continues throughout the infection. The roles of each IE protein in the pathogenesis of herpetic infections have been extensively investigated. ICP4 is required for viral growth. It acts as a DNA-binding protein, activating the majority of E and L genes, while suppressing certain IE genes through its interaction with basal transcription factors [65]. ICP22 is responsible for the expression of E genes and for some L genes, although the exact details of its mechanism of achieve this remains unclear, as well as the virion composition and egress from the nucleoplasm [66]. ICP27 is required for post-transcriptional modification of viral mRNA splicing, 3′ processing, and mRNA export [67,68,69]. The transport of peptides to the endoplasmic reticulum is prevented and blocked due to ICP47 binding to TAP1 or TAP2 [70]. In particular, the crucial role of ICP0 in the pathogenesis of HSV-1 infection is documented in the literature and has potential as a future therapeutic. Early studies have demonstrated its importance in productive infection [71], the establishment of a latent state, and successful reactivation [72]. Further studies have illustrated that ICP0 interacts with different cellular proteins to exhibit its functions. Everett et al. demonstrated that ICP0 locates at discrete subnuclear structures known as nuclear domain 10–ND10 [73,74] and leads to their disruption in a proteasome-dependent fashion [75]. This function was later attributed to the E3 ligase activity of the RING finger domain within the N-terminal segment of ICP0 protein [75].

### 2.4. Viral Early Phase

The E phase starts after the IE phase transcription genes have been activated, and it involves the replication of the viral genome. Viral DNA replication requires seven key replication proteins: pUL5, pUL8, and pUL52, forming the helicase/primase component; pUL9, the origin binding protein; pUL29 (ICP8), the single-strand DNA binding protein; pUL30, the viral DNA polymerase; and pUL42, the DNA polymerase processivity factor [76,77]. The synthesis of viral DNA is performed by the HSV-1 polymerase (pUL30) in combination with the pUL42 processivity factor [78,79,80]. The replication of viral DNA also stimulates the transcriptions of the L genes.

### 2.5. Late Phase

The final stage of the lytic cycle is the L phase, and it involves capsid envelopment, tegument formation, and egress of newly formed viral particles. The L phase genes are predominately expressed at the onset of the E phase during DNA replication; however, a subset termed leaky late or γ2 genes are formed prior to this. The assembly of newly synthetised capsids begins with the formation of the intermediate procapsid and takes place in the nucleus. The formation of a viral capsid is followed by DNA packaging that requires seven viral gene products: UL6, UL15, UL17, UL25, UL28, UL32, and UL33. The procapsid undergoes morphological change to become a mature icosahedral capsid and is released into the cytoplasm [81,82]. Initial studies have suggested that capsid envelopment takes place at the nuclear membrane. However, later studies have supported the double envelopment model, showing clear evidence of the capsid first losing its nuclear envelope before gaining its final envelope at the cytoplasmic membranes [83,84]. The site and sequence of tegument formation remains unclear, but it is presumed to largely occur in the cytoplasm. Some tegument proteins, described as the ‘inner’ tegument proteins (pUS3, pUL36, and pUL37), are thought to become incorporated directly onto capsids after exiting the nucleus [26]. However, the majority of tegument proteins, designated as ‘outer’ tegument proteins (pUL48 and pUL49), are believed to first associate with the viral glycoproteins at the site of virion envelopment at Golgi-derived vesicles [26]. Finally, the egress of viral particles occurs by the exocytosis of virus-containing vesicles [85].

The ability to establish an indefinite and latent infection in the sensory ganglia is a characteristic feature of HSV-1. During the latent phase, no viral particles or specific proteins are detectable, as the viral genome becomes associated with nucleosomes. The only viral genetic material detected during this phase are viral-specific mRNA molecules, or so-called LATs [86] The initial discovery that LATs are antisense to ICP0, a crucial viral regulatory protein, led to suggestions that LATs repress ICP0 expression therefore repressing a productive infection [87]. More recently, a murine oro-ocular model of herpetic infection suggests that LATs might be involved in the maintenance of HSV-1 latency through the post-transcriptional regulation of ICP0 in order to inhibit the expression of this potent activator of gene expression during latency [88]. Additionally, the absence of VP16 (normally required to initiate the immediate early viral gene expression) during the latent phase has raised questions about mechanisms of viral reactivation [89]. 

## 3. Host Cell–Virus Interaction

Innate immunity provides the foremost line of defence against both bacterial and viral infection, and it functions as an interface with the adaptive immune system. Following the transmission of HSV-1 by direct contact, the virus is initially suspended in the tear film, where it encounters a multi-pronged defence comprising enzymes, complement, immunoglobulins, and crucially, a range of antiviral and pro-inflammatory cytokines (interferon α, IL-12, TNFα, IL-6, amongst others) [90]. If it successfully penetrates the terminally differentiated outer corneal epithelial barrier, viral replication occurs within the deep epithelium and this is met with an influx of innate immune cells into the epithelium and anterior corneal stroma, including polymorphonuclear leucocytes, macrophages, and natural killer (NK) cells, which dominate the early inflammatory infiltrate and serve to eliminate the virus. A key pathogenic feature of HSV-1 relates to its ability to penetrate sensory neurons in the cornea and travel in a retrograde direction to the cell body in the trigeminal ganglion, where it remains latent, with the continued potential to reactivate, for the lifetime of the host.

In the majority of animal models studied, it was found that the live virus completely clears from the infected cornea within a week. Immune-mediated stromal keratitis developed seven to ten days post infection, which was characterised by the stromal infiltration of leucocytes, inflammation, oedema, opacification, and the growth of abnormal vessels in an otherwise avascular cornea [91]. Studies have demonstrated the importance of CD4 T cells in mediating stromal keratitis [92]. During the initial stages of HSK, neutrophils and CD4 T cells migrate from the limbus towards the site of corneal inflammation. Initially, CD4 T cell-mediated class II restricted cytotoxicity was suggested as a possible mechanism of immunopathology [93]. However, Niemialtowski and Rouse suggested that a more likely mechanism is a delayed type hypersensitivity response with some subset of CD4 T cells generating regulatory and inflammatory cytokines, which in turn may direct non-Ag-specific cells to exert tissue damage [94]. In their study, the authors demonstrated that Th1-type cytokines produced by CD4 T cells regulate neutrophil infiltration of the cornea and initiate the gradual growth of blood vessels into the cornea. These abnormal vessels act as a constant source of leucocytes and inflammatory cytokines, resulting in significant inflammation and corneal scarring. Therefore, whereas in most systems studied, Th1 cells appear to play an immune-protective role [95], in the case of HSK, the Th1 cells seem to be involved in a tissue-damaging immunopathologic reaction. Despite significant research into the role of CD4^+^ T cell-mediated responses in HSK, the exact mechanisms of CD4^+^ T cell activation within the cornea remain unclear. Research to date has failed to detect an active replicating virus in both animal models and human corneas, leaving the question of what causes CD4^+^ T cells to trigger the exact mechanisms of CD4 T cell activation within the cornea unanswered. Research to date has failed to detect an active replicating virus in both animal models and human corneas, leaving the question of what causes CD4 T cells to trigger HSK unresolved [96,97,98,99,100].

The detection of antigen-presenting cells (APC) in the cornea has opened the possibility that they play a role in HSV-1 immune responses. APCs such as dendritic cells (DC) and macrophages may acquire viral antigens from infected epithelial cells and subsequently trigger the immune response by presenting those antigens to naïve T cells in the lymph nodes. A HSK reactivation model has demonstrated a direct correlation between the number of DC in the cornea and the degree of persistent stromal opacification [101]. Another study showed that during bilateral infection of mice following a monocular depletion of DC cells, the HSK develops only in the non-depleted eye [102].

Some researchers have also suggested that autoimmunity can play a significant role in HSK pathogenesis. One of the mechanisms by which a virus can trigger an autoimmune response is molecular mimicry. There is evidence that supports the possibility of molecular mimicry between a common HSV-1 peptide, UL6, and a corneal protein that results in the production of autoreactive T cells [97].

One of the most rapid and critical immune responses to viral detection is the production of type I interferons (IFN-α and IFN-β) by infected cells. These work by inducing an antiviral state in both the infected and neighbouring cells, shutting down cellular transcription and translation, upregulating antigen-presenting molecules on their cell surface (MHC class I), and inducing the production of cytokines and chemokines that will attract natural killer cells and CD8 positive cytotoxic T cells. Cells have evolved a complex network of antiviral detection systems that, once activated, induce the synthesis and release of IFNs. Insights into how the innate immune system senses microorganisms have been provided with the discovery of the Toll-like receptors (TLR) [103]. In humans, the TLR family has 10 members that recognise conserved molecular motifs composed of proteins, carbohydrates, lipids, or nucleic acids, which are typically required for the survival or pathogenicity of the pathogen (pathogen-associated molecular patterns, or PAMPs). TLRs are highly expressed on corneal and conjunctival epithelial cells, as well as on antigen-presenting cells such as dendritic cells and macrophages [104]. After pathogen recognition, TLRs trigger intracellular signalling pathways that result in the induction of inflammatory cytokines, type I interferon, and chemokines. Broadly speaking, two types of viral PAMPs are recognised by TLRs: viral proteins and viral nucleic acid (DNA and RNA) [105].

A subfamily of TLRs (TLR3, 7, 8, and 9) are critical for viral detection by the innate immune system, and they have evolved to recognise viral nucleic acids. These have been dubbed the ‘Viral Tolls’ and are located in endosomal compartments. TLRs 3 and 7/8 recognise double-stranded RNA (dsRNA) and single-stranded RNA (ssRNA), respectively, whereas TLR9 recognises the dsDNA of HSV-1. It has also been suggested that HSV has TLR2-binding activity in the central nervous system, although this has not been demonstrated in the cornea [106]. Once activated, these pathogen recognition receptors (PRRs) participate in building an immune response appropriate to the pathogen encountered. Upon the detection of PAMPs, TLRs recruit downstream adaptor proteins such as myeloid differentiation primary response protein 88 (MyD88), MyD88 adaptor-like protein (Mal), Toll/interleukin 1 receptor domain-containing adaptor protein (TIRAP), Toll/interleukin 1 receptor domain-containing adaptor-inducing IFN-β (TRIF), and/or TRIF-related adaptor molecule (TRAM) [107,108]. This leads to the formation of protein complexes, including TANK-binding kinase 1 (TBK1) and inducible inhibitor of kB kinase (IKK) as well as the activation of downstream kinase cascades. This ultimately regulates the activation of transcription factors NF-κB and the interferon regulatory factor (IRF3, 5, and 7) family, and the consequent production of pro-inflammatory cytokines (TNFα and IL-12) and type I interferons, respectively. IRF3 is involved in the initial induction of IFN-β downstream of TLR4 and TLR3, which both recruit the adaptor protein TRIF in order to activate this pathway [109]. Evidence indicates that the kinases TBK1 and inhibitor of NFκB kinase-epsilon (IKKε) specifically phosphorylate serine residues in the C terminal domain of IRF3 and thereby activate it. IRF7 is similarly regulated by phosphorylation of C terminal serine residues, and IKKε and TBK1 are the kinases responsible.

MyD88 is a crucial adapter protein in most TLR signalling pathways, except TLR3 [110,111]. A model for intranasal HSV-1 infection showed that MyD88 knockout mice develop lethal encephalitis, whereas wild-type mice are resistant [112].

Another group of PRRs are the cytoplasmic RIG-I-like receptors (RLRs), which recognise dsRNAs. It is known that dsRNA is a by-product of HSV-1 replication and can therefore trigger RIG-I-regulated responses. RLRs are essential viral sensors in the cytoplasm and comprise Retinoic acid-inducible gene I (RIG-I), Melanoma differentiation-associated gene 5 (MDA5), and Laboratory of genetics and physiology 2 (LGP2), respectively [113]. Upon recognition of viral RNA, RIG-I and MDA-5 interact with the mitochondrial antiviral signalling protein (MAVS), which triggers the activation of a number of transcription factors such as IRF3 and NF-kB, leading to the production of IFNs and ISGs [114,115,116,117]. The connection between HSV and RIG-I was initially suggested by the observation that HSV-1 replicates robustly in human hepatoma cell lines lacking a functional RIG-I [118]. A recent study has demonstrated that US11, an RNA-binding tegument protein of HSV-1, binds to endogenous RIG-I and suppresses the downstream activation of the RLR signalling pathway, resulting in an inhibition of IFN-β [119]. Furthermore, studies have shown a collaboration of TLRs and RLRs for triggering antiviral innate immune responses [120,121,122]. Both pathways share crucial signalling factors, such as NF-kB, TBK1, IRF3, and IRF7, which have already been implicated in the complex interplay between HSV-1 and hosts’ antiviral responses [123].

Our understanding of innate immunity in HSK has been almost exclusively based on animal models of HSK. As humans are the only host for HSV-1 infection, defining our understanding of this disease using a mouse model is problematic. There are a limited number of human studies in this area that focus on TLR expression in human corneas with active and inactive HSK [124]. TLR expression was determined by Jin et al. in healthy donor corneas and HSV-1 infected corneas following corneal transplantation. They demonstrated an upregulation of all 10 TLRs in active HSK, especially TLR4, 7, 8, and 9, but only TLR7 in quiescent HSK. Two studies of human corneal epithelial cell lines and primary cell cultures have helped elucidate the role of TLR3, 7, and 9 in virus recognition; however, the molecular events of HSV-1 infection in the human cornea needs to be explored further to determine the potential to exploit these for the development of novel therapies for HSK, since it has not been investigated [125,126].

**Figure 4:**
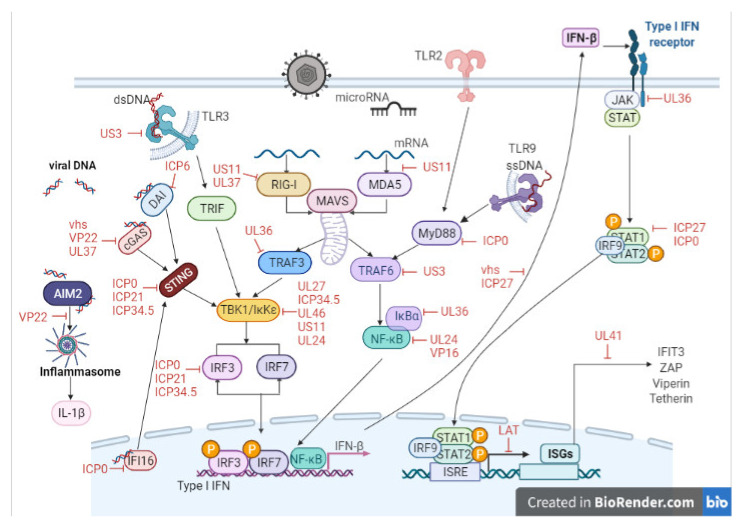
Schematic showing host cell–virus interactions leading to the initiation of an antiviral immune response. Red text indicate points on these pathways targeted by HSV-1 as part of its viral immune evasion strategy. Created with BioRender.com (https://biorender.com/, accessed on 1 July 2021).

More recently, several cytosolic DNA sensors have also been identified, including DNA-dependent activator of IFN regulatory factors (DAI), interferon gamma inducible protein 16 (IFI16), RNA polymerase III (Pol III), DEAD box helicase 41 (DDX41), and cyclic GMP-AMP synthase (cGAS), which contribute to the initiation of a host immune response upon the detection of viral nucleic acids [127]. cGAS, IFI16, and DDX41 signal through a common adaptor molecule known as Stimulator of IFN genes (STING). STING functions to recruit and activate TBK1, culminating in the activation of IRF3 and the induction of type I IFNs (IFN-I) (Figure 4).

During evolution, many viruses developed mechanisms to evade host responses and specifically the production of IFN-I by targeting different components downstream of the PRRs and cytosolic nucleic acid receptors [128]. IFN-I is essential to limit HSV-1 replication in the cornea as well as being required to limit the systemic spread of infection [129], and HSV-1 has evolved multiple strategies to evade the host immune response in order to establish latency [130,131].

## 4. Key Viral Proteins

There has been significant interest in determining the mechanisms used by HSV-1 to regulate IFN production given the central role it plays in limiting HSV-1 replication in the cornea as well as the systemic spread of infection [129]. Numerous HSV-1 viral proteins have been identified that contribute to evasion of the host immune response through an array of mechanisms. Figure 4 provides a summary of these antiviral evasion mechanisms that target the TLR signalling pathway, RLR signalling pathway, and DNA sensor signalling pathway, thereby inhibiting NF-κB activation (UL24, UL42, UL36) [132,133,134], modulating IRF3 (US3, VP16) [135,136], or STING (VP22) [137] function and consequently IFN-β production.

The HSV-1 encoded protein, infected cell protein 0 (ICP0), has been studied extensively in this regard, as it has been implicated in the pathogenesis of HSV-1 infection. ICP0 is a nuclear phosphoprotein that plays a crucial role in multiple aspects of the viral life cycle, including the transactivation of HSV-1 gene expression [138], initiation of lytic infection [139,140,141] and establishment of [142], and reactivation from a latent viral state [72,143,144]. Infection of cultured cells with an IE gene-deficient HSV-1 mutant that does not encode ICP0 is known to lead to complete repression of the viral genome and establishment of a quiescent state, with the only method of reactivation being to reintroduce ICP0 by superinfection [145,146,147,148].

ICP0, an E3 ubiquitin ligase, has developed various mechanisms to avoid immune-surveillance and promote viral replication. Studies have revealed that ICP0 induces proteasome-dependent degradation of the promyelocytic leukemia protein (PML) and Sp100 (speckled, 100 kDa) components of nuclear bodies [73,74,149,150,151]. In turn, this drives the dissociation of other host nuclear proteins death domain-associated protein (hDaxx) and ATP-dependent helicase (ATRX) from the viral genome, preventing them from repressing viral gene expression [152]. More recently, the E3 ligase Really Interesting New Gene (RING) finger domain of ICP0 has been shown to be responsible for proteasome-dependent degradation of several cellular proteins such as Nuclear domain 10 (ND10) [153]. After entering the cell, HSV-1 is confronted with early host defence transcriptional repression machinery including the corepressor of RE1 silencing transcription factor (CoREST) complex [154]. Interestingly it was found that in the presence of ICP0, 50% of the histone deacetylases that function in the CoREST complex dissociate, preventing transcriptional repression and enabling immune evasion [60,155,156]. Multiple studies have also shown that ICP0 inhibits IFN-I production, resulting in diminished innate immune responses by inhibiting IRF3 regulated transcription [136,157] and by inhibiting tumor necrosis factor alpha-induced NF-κB [158], STING [123] and IFI16 [159] activation.

It has also been shown to decrease MyD88, which is an adaptor protein with an important function in TLR signalling pathway. This allows the virus to evade the immune system’s inflammatory response that is mediated by TLR2 [123]. ICP0 targets IFI16, causing it to become ubiquitinated [13].

Recent studies investigated the potential that IRF7 may be a target for HSV-1 to overcome the innate antiviral response given that increased levels of this transcription factor have been observed in corneas of patients with a history of HSK [160,161]. While the exact molecular mechanism for this increased expression is unclear [162], a study by Murphy et al. [163] found that the trigeminal ganglia of double-deleted IRF3/7−/− infected mice had significantly higher viral loads than wild-type or single knockout mice, which suggests a synergistic control of HSV-1 pathogenesis by IRF3 and IRF7. Shahnazaryan et al. found that ICP0 inhibits the production of IFN-I driven by IRF7, suggesting an additional immune evasion strategy for HSV-1CP0 [164]. This is in keeping with the importance of IRF7 as a key target for immune evasion strategies as with other herpes viruses including Kaposi’s sarcoma-associated herpesvirus (KSHV) and Epstein–Barr virus (EBV) [165,166].

ICP4 is an IE protein that is also regulated by miR-H2 and plays a role in the reactivation process [167]. ICP47 is an important IE viral protein that allows HSV-1 to avoid being targeted by cytotoxic cluster of differentiation 8 (CD8) positive T cells, thereby enhancing viral survival [51]. An additional key viral protein is ICP34.5, which is a neurovirulence factor that is needed for viral replication [168]. It can bind TBK1, which then causes the inhibition of IFN production [123]. It is targeted by miR-H3 and miR-H4 and is also involved in the establishment of latency [169]. UL36 is HSV-1′s biggest tegument protein. Its main role is to facilitate viral replication. It works by deubiquitinating TNF receptor associated factor 3 (TRAF3), which is essential for type 1 IFN signalling. This inhibits the production of IFN-β, which in turn blocks TBK1 from being recruited [123]. There is still a lot of work to be done when it comes to understanding the function of these proteins and how they allow the virus to avoid the immune system, but with further research, it is possible that they could be targeted for potential therapeutics.

## 5. Current Treatments

There is no cure available for HSV, so once a person has been infected with the virus, it will remain dormant in the trigeminal ganglion throughout their lifetime, with the potential to reactivate. There are currently no vaccines available to prevent the acquisition of HSV-1. At the moment, VZV is the only member of the *Herpesviridae* family against which a vaccine exists [51].

Systemic treatment options are generally favoured by clinicians over topical administrations due to their superior bioavailability, ease of administration, and avoiding surface toxicity. Current treatment options using antivirals such as acyclovir or other nucleoside analogues to control HSV-1 infection have limited efficiency, ultimately failing to terminate the infection beyond its dormant state. The leading treatment for HSV-1 infections is acyclovir, a purine nucleoside analogue that has the ability to inhibit viral replication. It can be given via a number of administration routes including topical, oral, and intravenous administration depending on the specific HSV-1 manifestation in the eye [170]. Infections of the eyelids, conjunctiva, and cornea are most commonly managed with topical applications of acyclovir, usually for 2–3 weeks. Multiple studies have shown the therapeutic benefit of acyclovir in treating ocular HSV-1 infections as well as using it prophylactically to prevent recurrent infections [171,172,173]. Acyclovir works by inhibiting DNA polymerase, which in turn inhibits the replication of the virus. HSV thymidine kinase phosphorylates the drug so that it becomes acyclovir monophosphate. Then, this is phosphorylated a further two times, which causes it to become active in the form of acyclovir triphosphate. It only acts on infected cells as the phosphorylation step does not occur in the absence of infection [51]. Acyclovir has a high affinity for the virally infected cells, which is beneficial as it prevents the healthy cells from being attacked as well [164,171]. It is more effective in this way than some of the other available drugs such as topical trifluridine, which is a pyrimidine nucleoside analogue used to treat HSK. This drug has also been associated with toxicity due to it requiring frequent administration throughout the day, usually 8–10 times a day [13]. However, one of the main disadvantages of acyclovir is the rare occurrence of drug resistance, particularly in patients who are immunocompromised, as well as causing renal toxicity at high systemic doses [174,175]. There are multiple analogues of acyclovir, such as valacyclovir, famciclovir, and ganciclovir, all possessing a similar mechanism of action, but with differing bioavailability and dosing regimens [176,177,178].

Often, these antiviral treatment options are given in combination with topical steroids, which may not suitable for long-term use due to their side effect profile. This is due to the fact that the use of topical steroids for an extended period of time is linked with a number of issues such as secondary glaucoma, infection, and cataracts [179,180,181]. This is a major limitation when it comes to treating HSK, which is often recurrent and requires extended and well as prophylactic treatment regimens. These treatment options fail to eradicate the HSV-1 infection. Patients remain at risk of recurrent episodes of reactivation, and subsequent corneal opacification and scarring [182]. Corneal transplantation is the final option for recourse; however, these grafts are considered ‘high risk’ due to their failure rate in the context of previous HSV-1 infection [182].

## 6. Novel Treatments

### 6.1. Nucleic Acid Aptamers

Nucleic acid aptamers are single-stranded oligonucleotides that can bind to a wide range of molecular targets such as viral glycoproteins and cellular proteins with a high level of specificity [183]. They tend to be short and range in size from around 20 to 100 nucleotides. Their structural and functional diversity are what makes them good candidates for novel therapeutics for a number of diseases. They are capable of folding into various conformations, which is useful for binding to targets. Another benefit to aptamers is that they do not cause toxicity, and they work well at small concentrations [51]. However, they do have some disadvantages such as their high manufacturing costs and low stability [183]. A small number of studies have been carried out, and the results have shown that aptamers may have potential as a novel treatment for HSK. One of these studies demonstrated how using aptamers to bind the glycoprotein D (gD) protein of HSV-1 can inhibit viral entry into the host cells. The gD protein contains an ectodomain that is involved in the viral entry to the cells by coreceptor binding. This study involved two RNA aptamers, aptamer-1 and aptamer-5, that could bind the gD protein effectively. They both bind specifically to the HSV-1 gD protein with high affinity. This specificity is what gives the aptamer its ability to distinguish between the gD protein of HSV-1 and the gD protein of HSV-2 [184]. Another study was carried out that involved investigating the potential of aptamers against HSV-2, with the hope that by preventing HSV-2, it would in turn reduce HSV-1 morbidity. This is due to HSV-2 being considered a risk factor for HSV-1. The target in this study was also the gD protein, as it is essential for viral entry. The results showed that RNA aptamers can reduce HSV-2 infection when bound to the gD protein [185]. While the results of these studies are promising, designing aptamers against HSV-1 is still a relatively new area of research, so further studies are needed to fully define the benefits and risks of using them to treat patients who have been infected with HSV-1.

### 6.2. Toll-like Receptors

TLRs are transmembrane receptors that are involved in innate immunity. They are members of a family of receptors called pattern recognition receptors [186]. They are composed of three main sections: the ligand binding domain, a transmembrane domain, and a Toll/interleukin-1 receptor (TIR) domain [187]. This TIR domain facilitates the activation of downstream signalling pathways. TLRs play a role in the recognition and response to pathogens [114]. Due to their role in bridging the gap between the innate immune system and the adaptive immune system, they have the potential to be used in antiviral therapeutics, including treatment for HSV-1 infections [187]. Research has shown that various TLRs are involved in HSV infections [188], for example, there is evidence that TLR2 plays a role in the innate immune systems response to the virus. Using murine models of HSV-1 infection in wild-type (WT) and TLR2 knockout (KO) mice, Kurt-Jones et al. found that TLR2 mediates the induction of inflammatory cytokines in response to HSV-1 [106]. However, lethal viral encephalitis was observed in mice lacking TLR2 compared to WT mice, suggesting that TLR2 expression is not protective during HSV-1 infection [106].

Sarangi et al. found that mice lacking certain TLRs had fewer ocular lesions, their studies agree with the finding of Kurt-Jones et al., and they conclude that TLR responses during HSV-1 infection initiate disease pathology rather than providing a protective response in mouse models of disease [188]. Specifically, Sarangi et al. found that following HSV-1 infection, mice lacking TLR2 or TLR9 had fewer ocular lesions lesion compared to WT mice; however, these TLR-deficient mice had higher rates of lethal encephalitis [188].

In contrast, mice lacking TLR4 developed ocular lesions more rapidly than WT mice. Additionally increased levels of pro-inflammatory cytokines and angiogenic factors were observed in the corneas of TLR4-deficient mice compared to TLR2-deficient mice. The finding of reduced IL-10 levels in TLR4-deficient mice supports the suggestion that TLR2 and 9 may be driving ocular pathology during HSV-1, while TLR4 may play a more protective role [188].

Studies of TLR expression in human corneas during HSV-1 infection are limited. There is some evidence to suggest that similar to murine models, HSV-1 infection of human corneas increased the expression of TLRs [124]. Further studies in cell lines have shown that TLR3, 7, and 9 play roles in viral recognition [125,126]. While research in humans samples is limited, evidence from investigations in human cells lines have shed some light on the contribution of TLRs during HSV-1 in human cells. Of note, TLR2 signalling was shown to be activated by the HSV-1 glycoproteins gL and gH, which is of interest given that TLR2 is also responsible for the activation of NF-κB. This activation causes inflammatory cytokines such as interleukin 8 (IL-8) and IL-1 to be produced. A recent study investigated the connection between the gB glycoprotein encoded by HSV-1 and TLR2. The results of the study showed that this glycoprotein was recognised by TLR2 as a molecular target in human cell lines. This interaction suggests that there may be potential for using TLR2 in therapeutics, as it may allow early recognition of the HSV-1 glycoprotein by the innate immune system, possibly before the virus has entered the host cells [186].

Given the adverse immune consequences associated with TLR activation, especially TLR2 and TLR9 for neovascularisation and lesions at the ocular surface, significantly more research is required in both animal and human models to fully understand the role played by these innate immune sensors during HSV-1 viral entry and infection. TLR-based therapeutics have the potential to enhance the immune response during HSV-1 infection; therefore, therapeutics that modulate their function need to balance the protective versus the pathologic effect. New treatments that alter TLR responses at the ocular surface with locally acting compounds with idealised half-life offer potential here.

### 6.3. Antibodies

The pathogenesis of HSV-1 involves a complex interaction between cytokines, chemokines, and growth factors, either brought in by inflammatory cells or produced locally. Avoidance of these innate antiviral responses can cause lifelong recurrent infection, which in turn can cause progressive corneal scarring, vascularisation, thinning, and the need for corneal transplantation to recover vision, often with a poor long-term outcome. We conducted a study in HSK patients with active and inactive infection to investigate peripheral cytokine production in order to identify potential therapeutic targets.

We determined that patients with active corneal HSV-1 infection had significantly elevated serum levels of the pro-inflammatory cytokine IL-1β compared to healthy controls [189]. Furthermore, IL-1β levels remained significantly increased in these patients following treatment. Elevated production of IL-1β in inactive patients was associated with significantly increased levels of IRF3 and STAT1, which are two key proteins involved in promoting antiviral immune responses [189]. Taken together, our data suggest that enhanced peripheral production of the pro-inflammatory cytokine IL-1β may have implications for HSV-1 viral clearance in active and inactive HSK patients. The finding that elevated levels of IL-1β persisted beyond the period that was clinically evident suggests that enhanced peripheral production of the pro-inflammatory cytokines IL-1β and antibodies against IL-1β may have implications for HSV-1 viral clearance.

In support of this, murine studies suggest that cytokines may be important contributors to the development of HSK pathogenesis. These studies have shown that following HSV-1 infection of the cornea, the most prominent cytokines that are produced are IL-6 and IL-1, typically several days after the development of stromal keratitis. This has been shown in murine models in vivo and in excised mouse corneas [190,191]. Subsequent studies have shown that elevated levels of IL-1β and TNF-α are associated with corneal inflammation, while IL-6 and TGF-β are thought to exert antiviral and inflammation regulatory activities in HSV-1 corneal infection [192]. More recent studies have confirmed that elevated levels of IL-1β and TNF-α induced by HSV-1 infection, in the context of microglial cells at least, are not important for inhibiting viral replication but instead play a role in the pathogenesis of HSV-1 infection [193]. IL-1 has been shown to promote the production of IL-17, and recent studies have implicated this cytokine in the pathogenesis of HSK [194]. These studies found that following HSV-1 infection, IL-17 was detected in infected corneas, and its suppression reduced the severity of the HSK [194,195]. While current evidence supports a role for cytokines acting locally in the cornea, the precise role of peripheral cytokines have not yet been characterised. Thus, monitoring the levels of these cytokines in the periphery might prove to be a useful diagnostic tool for predicting relapses of HSK in addition to providing novel treatment options.

Previous investigations have shown that prophylactic oral dosing with antivirals reduced the recurrence of ocular HSK consequently, current treatments for HSK include topical and systemic antiviral drugs such as acyclovir and trifluorothymidine [196]. Thus, targeting IL-1β locally and in the periphery may potentially have a beneficial outcome for localised keratitis induced by HSV-1. In support of this, recent studies have shown that mice transgenic for the IL-1 receptor antagonist protein are resistant to HSK [197]. Further studies are required to determine if therapies used in the treatment of inflammatory disorders characterised by the overproduction of IL-1β, such as anakinra, a recombinant IL1-Ra antibody, and canakinumab, an anti-IL-1β monoclonal antibody (mAb), hold potential for the treatment of HSK [198,199,200]. Given the central role HSV-1 glycoproteins have in mediating viral entry and the induction of an immune response, a considerable amount of research has involved the generation and characterisation of antibodies against HSV-1 glycoproteins.

One such study was carried out in 2013 to investigate if humanised monoclonal antibodies (mAbs) could be used to neutralise HSV-1 and HSV-2 infections. The results of this study showed that the mAb hu2c was capable of neutralising HSV-1 [201]. Furthermore mAb hu2c was shown to be more efficient against HSV-1 than it was against HSV-2, as higher concentrations of mAb were needed to have an effect on HSV-2 [201]. Du et al. generated a novel virus-neutralising monoclonal antibody whose epitope was located within a continuous antigenic determinant. They reported that the mAb m27f inhibited an important immune evasion mechanism of HSV-1: cell-to-cell spread [202]. The mAb binds to the prodomain, which is a region of the gD protein that is highly conserved. This domain is responsible for the binding of the virus to the cell, so it is necessary for viral entry. This is the reason why the gD protein is a good target to choose when it comes to novel therapeutics and vaccine development. By blocking the gD receptor, the mAb ensures the virus is unable to enter the host cells [203].

While strategies that target viral glycoproteins may hold promise, care should take when selecting glycoprotein subunits for inclusion in vaccines or as determinants for monoclonal antibodies [204].

Since 1994, Ghiasi et al., have generated recombinant baculoviruses expressing high levels of known HSV-1 glycoproteins and tested their vaccine efficacy against that of primary ocular HSV-1 challenge in mice. Overall, these investigations have categorised 10 baculovirus-expressed genes into four groups and reported that: (i) Immunisation with gB, gC, gD, gE, or gI completely protects mice against lethal challenge; (ii) No significant protection was seen with gH, gJ, and gL; (iii) Immunisation with gK leads to severe exacerbation of eye disease and scarring; and (iv) Immunisation with gG also showed a tendency to be harmful [205].

There are some disadvantages associated with antibody therapy including high cost and the long generation process [206]. Overall, these studies show potential for the use of antibodies as therapeutics, but further research is needed in this area, particularly to study the risks and the effects associated with using these antibodies in humans.

### 6.4. MicroRNA

MiRNAs are small non-coding molecules of RNA. They are 18–22 nucleotides in length and are involved in the process of regulating gene expression [167]. The human genome encodes more than 2000 miRNAs, which regulate the expression of approximately 60% of the genes for protein coding [22]. They play a part in a large number of cellular processes, including homeostasis and development. Their role in viral reactivation, particularly in the case of HSV-1, has been an area of interest in recent years [169]. If the involvement of miRNAs in viral processes can be studied further and fully understood, it is thought that more effective novel treatments can be designed using them as targets [207].

In addition to forming part of the strategy used by HSV-1 to enter cells and maintain latency, HSV-1 infection of cells causes significant alterations in the expression of host microRNAs [207,208]. These miRs regulate an array of host and viral transcripts, ultimately feeding into several key processes including the regulation of apoptosis, antiviral immunity, and inhibition of viral replication [207]. Furthermore, Pan et al. suggest that in addition to subverting the immune response, HSV-1 utilises host microRNA to establish latency and promote viral spread throughout the population [209].

Initial studies identified several viral microRNAs including miR-H1, which is highly expressed during productive infection [210], and miR-H2-H6, which is elevated in latency. Umbach et al. demonstrated that miR-H2-3p can reduce ICP0 protein expression while miR-H6 inhibits the expression of ICP4 (which is required for the expression of most HSV-1 genes during productive infection) [208]. Pan et al. found that the neuronal expression of miR-138 can repress HSV-1 lytic gene expression, promoting host survival and viral latency [209]. It does so by repressing the expression of the HSV-1 lytic gene transactivator, ICP0.

HSV-1 has its own miRNAs such as miR-H1 and miR-H2, which allow it to create a more hospitable environment and prolong its survival. They can also aid in avoiding the host immune response; for example, there is evidence that miR-H27 plays a role in this process along with the proliferation and replication of the virus. Host miRNAs have antiviral properties and are thought to be involved in the establishment of latency. One example of this is miR-101, which targets the 3′ untranslated region of a key protein, mitochondrial adenosine triphosphate (ATP) synthase subunit beta. This leads to the downregulation of viral replication, which may be one of the many contributing factors in establishing latency [169]. MiR-23a is involved in a large number of biological pathways, which include fatty acid biosynthesis, the thyroid hormone signalling pathway, and the cell cycle. It carries out its function by modulating its target genes, which include zinc finger protein 138 (ZNF138), semaphorin 6D (SEMA6D), and TGF-beta activated kinase 1 (MAP3K7) binding protein 3 (TAB3). It is also thought to play a role in a number of cancers if it becomes dysregulated, particularly acute myeloid leukaemia and small cell lung cancer [211]. Research into miR-23a has shown that it can inhibit an antiviral innate immune pathway, the IFN pathway, by targeting IRF1. In turn, this allows the replication of HSV-1 to occur. Therefore, this could be a potential target for therapeutics. By blocking this interaction, the antiviral pathway may be able to proceed and inhibit HSV-1 replication [169]. MiR-132 is involved in a number cellular pathways such as the TGF-beta signalling pathway, the cell cycle, and the hippo signalling pathway. It has been shown to play a role in the viral replication process in a number of viruses, including HSV-1. Studies carried out on mice have shown that miR-132 is upregulated in the cornea post HSV-1 infection. This indicates that it plays a role in the infection [207]. MiR-21 is another interesting miRNA that plays a role in a large number of pathways. Some of these include the prolactin signalling pathway, sphingolipid metabolism, the p53 signalling pathway, and fatty acid degradation. Its main functions in the cell are to stimulate angiogenesis and cellular migration [179]. It is also involved in the inhibition of apoptosis [207]. These roles indicate that miR-21 may be involved in cancer processes [169]. It has been shown to be upregulated in a number of cancers such as endometrial and colorectal cancer [212]. Studies in mouse models have indicated that miR-21 is overexpressed in mice with Behcet’s disease after HSV-1 infection. With further studies, the significance of this overexpression may become more understood, which could lead to potential therapeutic opportunities for miR-21 [207]. MiR-146a is a miRNA that is involved in cell proliferation and the regulation of T cell differentiation [169]. It is mainly expressed in monocytes where it is involved in pathways such as the NF-κB signalling pathway and the TLR signalling pathway, in addition to having a role in the cell cycle [22]. When overexpressed, it potentially causes interleukin 1 receptor-associated kinase 1 (IRAK1) to become downregulated. This has a knock-on effect for the NF-κB signalling pathway, disrupting its ability to promote inflammation via pro-inflammatory cytokines [203]. It is one of a small group of miRNAs that are commonly associated with viral infections, and it has been shown to be overexpressed in HSV-1 infections [207]. It promotes the arachidonic acid cascade by binding complement factor H, which then allows HSV-1 to avoid being targeted by the immune system [169]. Due to this, miR-146a may be a potential target for a novel treatment against HSV-1 infections.

While some miRs are important for subverting the induction of effective anti-viral immune responses, others play key roles in promoting viral latency. A greater understanding of the role played by these miRs has the potential to lead to the generation of novel targeted treatments for HSK. Therapeutic strategies to modulate miR expression will utilise synthetic oligonucleotides sequences that can increase (miR-mimic) or reduce (antagomir) miR expression. The feasibility for this approach has been shown in the context of hepatitis C virus (HCV) [213]. miR-122 has been shown to be essentially required for the stability and replication of HCV, leading investigators to develop a therapeutic Miravirsen, which inhibits miR-122 function [214]. In clinical trials, Miravirsen (NCT01200420) was shown to reduce HCV RNA without causing viral resistance in patients with chronic HCV genotype 1 infection [214]. Thus, miR-based treatments may hold promising outcomes for patients susceptible to recurrent HSV-1 infection.

## 7. Conclusions

HSV-1 infection is highly prevalent throughout the world, and despite advances in our understanding of its pathogenesis and treatment, ocular infections carry a high risk of sight loss. It is evident that there is a need for novel treatments for ocular HSV-1 as the current treatments, such as acyclovir, are not completely effective in recurrent infections and known to be affected by drug resistance. In addition, one of the most effective treatments, topical corticosteroids, is often not suitable for long-term use because of serious and common adverse effects. There is also a need to identify and develop prognostic and diagnostic biomarkers for clinical use, as this could facilitate earlier intervention and better prophylactic management of appropriate patients.

## Data Availability

Not applicable.

## List of Abbreviations

APC	Antigen-presenting cell
3-OS-HS	3-O-sulfated heparan sulfate proteoglycan
ATP	Adenosine triphosphate
ATRX	ATP-dependent helicase
CHO	Chinese hamster ovary
CD8	Cluster of differentiation 8
CoREST	Corepressor of RE1 silencing transcription factor
cGAS	cyclic GMP-AMP synthase
DDX41	DEAD box helicase 41
hDaxx	Death domain-associated protein
DC	Dendritic cells
DNA	Deoxyribonucleic acid
DAI	DNA-dependent activator of IFN regulatory factors
dsRNA	Double-stranded RNA
E	Early
ELISA	Enzyme-linked immunosorbent assay
EBV	Epstein–Barr virus
g	Glycoprotein
HSPGs	Heparan sulfate proteoglycans
HCV	Hepatitis C virus
HSK	Herpes stormal keratitis
HSV-1	Herpes Simplex Virus type 1
HSV-2	Herpes simplex virus type-2
HVEM	Herpesvirus Entry Mediator
pUL30	HSV-1 polymerase
pUL29	ICP8
IE	Immediate Early
ICP	Infected cell proteins
IKK	Inhibitor of kB kinase
IFI16	Interferon gamma inducible protein 16
IRF	Interferon regulatory factor
IFN-α	Interferon-α
IFN-β	Interferon-β
IL	Interleukin
IRAK1	Interleukin 1 receptor-associated kinase 1
KSHV	Kaposi’s sarcoma-associated herpesvirus
KO	Knockout
LGP2	Laboratory of genetics and physiology 2
LAT	Latency Associated Transcript
L	Latency Associated Transcript
MDA5	Melanoma differentiation-associated gene-5
MAVs	Mitochondrial antiviral signalling protein
mAB	Monoclonal antibody
Mal	MyD88 adaptor-like protein
MyD88	Myeloid differentiation primary response protein 88
NK	Natural killer
IKKε	NFκB kinase-epsilon
ND10	Nuclear domain 10
PAMPs	Pathogen associated molecular patterns
PRR	Pathogen recognition receptors
PCR	Polymerase chain reaction
PML	Promyelocytic leukemia protein
RING	Really Interesting New Gene
RIG-I	Retinoic acid inducible gene-I
RLRs	RIG-I-like receptors
Pol III	RNA polymerase III
SEMA6D	Semaphorin 6D
ssRNA	Single-stranded RNA
Sp100	Speckled, 100 kDa
STING	Stimulator of IFN genes
TBK1	TANK-binding kinase 1
TAB3	TGF-beta activated kinase 1 (MAP3K7) binding protein 3
TRAF3	TNF receptor-associated factor 3
TLR	Toll-like receptors
TIRAP	Toll/interleukin 1 receptor domain-containing adaptor protein
TRIF	Toll/interleukin 1 receptor domain-containing adaptor-inducing IFN-β
TIR	Toll/interleukin-1 receptor
TRAM	TRIF-related adaptor molecule
IFN-I	Type 1 IFN
VZV	Varicella-zoster virus
UL41	Virion-induced host shutoff protein, VHS
WT	Wild type
ZNF138	Zinc finger protein 138
